# Myogenic-specific ablation of Fgfr1 impairs FGF2-mediated proliferation of satellite cells at the myofiber niche but does not abolish the capacity for muscle regeneration

**DOI:** 10.3389/fnagi.2015.00085

**Published:** 2015-05-28

**Authors:** Zipora Yablonka-Reuveni, Maria E. Danoviz, Michael Phelps, Pascal Stuelsatz

**Affiliations:** Department of Biological Structure, University of Washington School of Medicine, SeattleWA, USA

**Keywords:** satellite cells, fibro/adipogenic progenitors, fibroblast growth factor, Pax7, MyoD^Cre^, alpha7 integrin, cardiotoxin injury, muscle spindles

## Abstract

Skeletal muscle satellite cells (SCs) are Pax7^+^ myogenic stem cells that reside between the basal lamina and the plasmalemma of the myofiber. In mature muscles, SCs are typically quiescent, but can be activated in response to muscle injury. Depending on the magnitude of tissue trauma, SCs may divide minimally to repair subtle damage within individual myofibers or produce a larger progeny pool that forms new myofibers in cases of overt muscle injury. SC transition through proliferation, differentiation and renewal is governed by the molecular blueprint of the cells as well as by the extracellular milieu at the SC niche. In particular, the role of the fibroblast growth factor (FGF) family in regulating SCs during growth and aging is well recognized. Of the several FGFs shown to affect SCs, FGF1, FGF2, and FGF6 proteins have been documented in adult skeletal muscle. These prototypic paracrine FGFs transmit their mitogenic effect through the FGFRs, which are transmembrane tyrosine kinase receptors. Using the mouse model, we show here that of the four Fgfr genes, only Fgfr1 and Fgfr4 are expressed at relatively high levels in quiescent SCs and their proliferating progeny. To further investigate the role of FGFR1 in adult myogenesis, we have employed a genetic (Cre/loxP) approach for myogenic-specific (MyoD^Cre^-driven) ablation of Fgfr1. Neither muscle histology nor muscle regeneration following cardiotoxin-induced injury were overtly affected in Fgfr1-ablated mice. This suggests that FGFR1 is not obligatory for SC performance in this acute muscle trauma model, where compensatory growth factor/cytokine regulatory cascades may exist. However, the SC mitogenic response to FGF2 is drastically repressed in isolated myofibers prepared from Fgfr1-ablated mice. Collectively, our study indicates that FGFR1 is important for FGF-mediated proliferation of SCs and its mitogenic role is not compensated by FGFR4 that is also highly expressed in SCs.

## Introduction

Skeletal muscle is composed of multinucleated myofibers that are established during embryogenesis by fusion of myoblasts. Addition of myofiber nuclei (myonuclei) or formation of new myofibers during postnatal and adult life depend on satellite cells (SCs), Pax7^+^ myogenic progenitors that are localized between the basal lamina and the plasmalemma of the myofiber (Mauro, 1961; Seale et al., 2000; Yablonka-Reuveni, 2011). During postnatal growth, at least some SCs are proliferative and contribute progeny that fuse with the enlarging myofibers (Moss and Leblond, 1971; Schultz, 1996; Halevy et al., 2004; White et al., 2010). In mature muscles, SCs are typically quiescent, but can be activated in response to muscle injury (Schultz et al., 1978; Montarras et al., 2013). Depending on the magnitude of tissue trauma, SCs may divide minimally to repair subtle damage within individual myofibers or produce a larger progeny pool that forms new myofibers in cases of overt muscle injury (Grounds and Yablonka-Reuveni, 1993; Hawke and Garry, 2001). In addition to generating myogenic progeny that fortify myofibers, at least some SCs can self-renew, thereby meeting the defining criteria of bona fide resident stem cells (Collins et al., 2005; Day et al., 2007; Kuang et al., 2007; Sacco et al., 2008).

At the molecular level, SCs and their progeny are tightly regulated by highly orchestrated temporal expression of transcription factors and cell cycle regulators, providing a balance between SC quiescence, proliferation, differentiation and renewal (Bentzinger et al., 2010; Yablonka-Reuveni and Day, 2011; Yin et al., 2013). To monitor progression through these stages, researchers have relied on distinct marker signatures, in particular, temporal expression of the paired box transcription factor Pax7, and the myogenic regulatory factors MyoD and myogenin (Yablonka-Reuveni and Rivera, 1994; Zammit et al., 2006; Yablonka-Reuveni et al., 2008; Yablonka-Reuveni, 2011). Proliferating progeny maintain Pax7 expression as their quiescent progenitors, but distinctly, are also MyoD-positive (Zammit et al., 2004). A decline in Pax7, along with the induction of myogenin, marks progeny that have entered into the differentiation phase and subsequently may fuse into myotubes (Shefer et al., 2006; Day et al., 2009). Re-emergence of cells that express Pax7, but not MyoD, defines a self-renewing population of SCs known as reserve cells (Halevy et al., 2004; Zammit et al., 2004; Day et al., 2007).

Satellite cell transition through proliferation, differentiation and renewal is not only governed by the molecular blueprint of the cells, but is also regulated by the extracellular milieu at the SC niche (Allen et al., 1984; Allen and Boxhorn, 1989; Anderson, 2006; Brack and Rando, 2007; Shefer and Yablonka-Reuveni, 2008; Yin et al., 2013; Wang et al., 2014). Isolated myofibers maintained in conditions where the SCs and their progeny are retained at their native position, have offered a unique *in vitro* means to investigate the effect of growth factors on SC behavior at their native niche (Bischoff, 1986a; Yablonka-Reuveni and Rivera, 1994; Yablonka-Reuveni et al., 1999a). Using this approach, hepatocyte growth factor (HGF) and selective members of the fibroblast growth factor (FGF) family have been shown to enhance SC proliferation (Bischoff, 1986a,b; Yablonka-Reuveni et al., 1999a,b; Kastner et al., 2000; Wozniak and Anderson, 2007), while transforming growth factor beta (TGFβ1) has been found to repress proliferation (Bischoff, 1990; Yablonka-Reuveni and Rivera, 1997b). Our particular interest in the role of the FGFs and their receptors in regulating SC dynamics through life (Yablonka-Reuveni and Rivera, 1994, 1997b; Yablonka-Reuveni et al., 1999a,b; Kastner et al., 2000; Shefer et al., 2006; Kwiatkowski et al., 2008) has prompted the research described in the current study.

The FGFs are key players in the processes of proliferation and differentiation of a wide range of cells and tissues. Over 20 FGFs, classified as paracrine (FGFs 1–10, 16–18, 20, 22), endocrine (FGFs 15/19, 21, 23) and intracrine (FGFs 11–14) types, have been discovered to date (Mason, 2007; Itoh and Ornitz, 2011; Ohta and Itoh, 2014). Selective paracrine FGFs have long been known to act as mitogens of SCs [i.e., FGF1, FGF2, FGF4, and FGF6, but not FGF5, FGF7, and FGF8 (Sheehan and Allen, 1999; Kastner et al., 2000)]. Importantly, several of these paracrine FGFs that can promote SC proliferation (FGF1, FGF2, FGF6) have been detected at the transcript and the protein levels in adult skeletal muscle (Yamada et al., 1989; Alterio et al., 1990; Le Moigne et al., 1990; Oliver et al., 1992; Clarke et al., 1993; Dusterhoft et al., 1999; Kastner et al., 2000; Zhao and Hoffman, 2004; Fon Tacer et al., 2010; Chakkalakal et al., 2012). In particular, FGF2 (formerly known as basic FGF) has been used extensively as the FGF of choice in many studies of SCs in single myofibers (Yablonka-Reuveni and Rivera, 1994, 1997b; Yablonka-Reuveni et al., 1999a,b; Shefer et al., 2006) and as a routine medium supplement in primary cultures (Rando and Blau, 1994; Motohashi et al., 2014). Apart from its mitogenic effect, FGF2 has been suggested to directly repress myoblast differentiation, thereby supporting expansion of the proliferative pool (Clegg et al., 1987; Olwin et al., 1994).

Studying SCs in isolated myofibers under conditions that retain SCs at the myofiber niche, we previously showed that SCs from senile mice (29–33 months) could not enter a proliferative state without FGF2 supplementation, whereas SCs from young mice (3–6 months) did not require exogenous FGF2 (Shefer et al., 2006). In accordance with our findings, a recent study reported that FGF2 is required to remove age-associated proliferative inhibition of SCs (Li et al., 2014). We also demonstrated that an FGF2 activity-blocking antibody drastically reduced SC activation/proliferation in isolated myofibers from young rodents (Yablonka-Reuveni and Rivera, 1994). Collectively, our studies indicate that FGF2 is required for SC proliferation and that FGF2 (or FGF2-mediated signaling) becomes rate limiting in SC function in old age, and this may be an underlying factor in the age-associated decline in SC numbers observed in some limb muscles (Brack et al., 2005; Shefer et al., 2006, 2010, 2013). However, it has been reported that excess FGF2 harbored in the myofibers of aging mice leads to SC depletion due to detrimental proliferation (without self-renewal), rather than retention of the quiescent state (Chakkalakal et al., 2012). Hence, means for direct ablation of FGF2 signaling are needed to assist in determining its role in SC performance during aging.

As the paracrine FGFs mediate their biological responses by binding to cell surface FGF receptors (FGFR1, FGFR2, FGFR3, FGFR4), FGFR impairment offers one possible approach for studying the effect of FGF2 signaling on SC performance. The FGFRs share a common “generic” structure consisting of an extracellular region containing three immunoglobulin-like domains (Ig-1, Ig-2, Ig-3), a transmembrane domain, and an intracellular domain containing a tyrosine kinase core. FGF binding to the FGFR extracellular domain induces receptor dimerization and activation of the tyrosine kinase domain, which can initiate key downstream intracellular signaling pathways: RAS–RAF–MAPK, PI3K–AKT, STAT, and PLCγ (Eswarakumar et al., 2005; Mason, 2007; Lanner and Rossant, 2010; Goetz and Mohammadi, 2013). While the FGFRs are encoded by four separate genes (Fgfr1, Fgfr2, Fgfr3, Fgfr4), alternative splicing variants, alongside the temporal and spatial regulation of expressed FGF and FGFRs and the involvement of additional co-factors, increase the complexity and specificity of FGF signaling (Ornitz, 2000; Zhang et al., 2006; Mason, 2007; Itoh and Ornitz, 2011; Goetz and Mohammadi, 2013). Out of the four FGFRs, typically only FGFR1 and FGFR4 have been considered in the context of adult myogenesis, due to their relative higher transcript levels observed in freshly isolated SCs and myogenic cultures [(Sheehan and Allen, 1999; Cornelison et al., 2000; Kastner et al., 2000; Jump et al., 2009; Chakkalakal et al., 2012); current study]. Furthermore, to date only FGFR1 and FGFR4 have been documented at the protein level in SCs or their progeny (Cornelison et al., 2001; Kwiatkowski et al., 2008; Cassano et al., 2011). While our overexpression studies have suggested different modes of function for FGFR1 and FGFR4 (Kwiatkowski et al., 2008), it is unknown whether these two FGFRs can compensate for each other during SC myogenesis. Pharmacological-based abrogation of FGFR-signaling has been employed in order to elucidate the role of FGFR1 in the context of SC dynamics (Chakkalakal et al., 2012; Bernet et al., 2014). However, the inhibitory drug used, SU5402 (Mohammadi et al., 1997), can theoretically target all FGFRs based on its effect on blocking FGFR tyrosine kinase function. Indeed, SU5402 has been used as a general inhibitor of FGF signaling in different species regardless of the expressed FGFR (Udayakumar et al., 2003; Delaune et al., 2005; Dvorak et al., 2005; Abe et al., 2007; Thomsen et al., 2008; Vatsveen et al., 2009; Franzdottir et al., 2010; Fukui and Henry, 2011; Li et al., 2013). Myogenic-specific ablation or overexpression of Spry1, a member of the Sprouty family of negative regulators of receptor tyrosine kinase signaling (Cabrita and Christofori, 2008), were also employed to modulate FGF signaling during adult myogenesis (Chakkalakal et al., 2012). The Sprouty proteins, however, act as inhibitors of the Ras/MAPK cascade, a pathway downstream of various receptor tyrosine kinases beyond just the FGFRs (Mason, 2007; Cabrita and Christofori, 2008), which can complicate data interpretation.

If FGFR signaling is essential for regulating SC pool size, which in turn may be important for muscle homeostasis, then a better understanding of this topic is needed when considering future therapies for disease- or age-associated muscle wasting. Gaining further understanding of the role of the FGFR system in myogenesis requires models that facilitate direct FGFR ablation, bypassing downstream interventions that may not specifically target individual FGFRs and may affect additional tyrosine kinase receptor cascades. In the current study we have aimed to gain insight into the role of FGFR1 during adult myogenesis using Fgfr1-ablated mice. As standard Fgfr1-null mice die during gastrulation (Deng et al., 1994; Yamaguchi et al., 1994), investigations of the role of FGFR1 in fetal and adult life have only become possible with the development of conditional Fgfr1-null alleles (Xu et al., 2002; Trokovic et al., 2003). Here, we have ablated Fgfr1 specifically in the myogenic lineage using a genetic approach with a Cre/loxP mouse model that relies on the MyoD^Cre^ allele to mediate excision of the floxed Fgfr1 gene. MyoD is well recognized as a master regulator of the myogenic lineage specification during embryogenesis (Weintraub et al., 1991). While SCs are thought to express MyoD only upon their activation (Yablonka-Reuveni and Rivera, 1994; Cornelison and Wold, 1997; Yablonka-Reuveni et al., 2008), SC progenitors do emerge during embryogenesis from a MyoD-expressing lineage (Kanisicak et al., 2009; Yamamoto et al., 2009). Thereby, MyoD^Cre^-mediated excision of floxed genes would occur in the embryonic muscle and be stably maintained in the myogenic lineage through adult life. Here we show that myogenic-specific ablation of Fgfr1 does not appear to influence muscle morphology or regeneration following cardiotoxin-induced damage in adult mice. Nevertheless, our study provides novel evidence for the obligatory role for FGFR1 in mediating FGF2 mitogenic effect on SCs that is not compensated by FGFR4, which is also highly expressed in SCs.

## Materials and Methods

### Mice

Experimental procedures were approved by the University of Washington Institutional Animal Care and Use Committee. Mice were typically 4–6 months of age. Knockin heterozygous males MyoD^Cre^ [MyoD1^tm2.1(icre)Glh^ (Kanisicak et al., 2009)] provided by David Goldhamer, backcrossed by us to C57BL/6, were bred with knockin reporter females R26^mTmG^ [Gt(ROSA) 26Sor^tm4(ACTB-tdTomato,-EGFP)Luo^/J (Muzumdar et al., 2007)] to generate adult F1 MyoD^Cre/+^/R26^mTmG/+^ double heterozygous animals. Mice harboring floxed Fgfr1 alleles (Trokovic et al., 2003) were provided by David Ornitz (White et al., 2007). These mice additionally harbored floxed FGFR2 (Yu et al., 2003). Nevertheless, as discussed in the Introduction, FGFR2 has been considered not relevant in adult myogenesis and indeed, as shown in Results, Fgfr2 transcript expression in SCs and their progeny is negligible. The Fgfr1^fl/fl^/Fgfr2^fl/fl^ females were crossed with MyoD^Cre/+^/R26^mTmG/+^ males and the resulting MyoD^Cre/+^/R26^mTmG/+^/Fgfr1^fl/+^/Fgfr2^fl/+^ males were backcrossed with Fgfr1^fl/fl^/Fgfr2^fl/fl^ females to produce MyoD^Cre/+^/R26^mTmG/+^/Fgfr1^fl/fl^/Fgfr2^fl/fl^ experimental animals harboring muscle-specific (i.e., MyoD-driven) Fgfr deletions. The FGFR1^fl^ allele contains loxP sites flanking exons 8–15 that encompass the transmembrane domain and most of the intracellular region (Trokovic et al., 2003). The FGFR2^fl^ allele contains loxP sites flanking exons 8-10 that encode a portion of the ligand binding Ig-3 domain and the transmembrane domain (Yu et al., 2003).

Primers for genotyping the MyoD^Cre^ (JAX mice stock #014141) and R26^mTmG^ (JAX mice stock #007676) alleles were according to Jackson Lab. Primers for genotyping the floxed Fgfr alleles were according to (Trokovic et al., 2003; White et al., 2007). Myogenic specificity of the MyoD^Cre^-driven Fgfr deletions was confirmed by the detection of Fgfr delta alleles (Fgfr1^Δ^, Fgfr2^Δ^) only in skeletal muscles but not in other control organs; PCR primers were according to (Trokovic et al., 2003; White et al., 2007). Likewise, GFP fluorescence was detected only in skeletal muscle myofibers and SCs as we previously published for MyoD^Cre/+^/R26^mTmG/+^ mice (Stuelsatz et al., 2012, 2014).

Mice carrying a MyoD-null allele (Rudnicki et al., 1992) or α7integrin-null allele (Flintoff-Dye et al., 2005) in a heterozygous or homozygous format were additionally used for comparison when analyzing SC numbers in isolated myofibers from Fgfr1/Fgfr2-ablated mice. Both null strains were utilized in our earlier studies (Yablonka-Reuveni et al., 1999a; Kirillova et al., 2007; Rooney et al., 2009; Stuelsatz et al., 2012) and genotyped according to published procedures (Valdez et al., 2000; Flintoff-Dye et al., 2005). Apart from the MyoD^+/-^ and MyoD^-/-^ mice that were on Balb/C background, all other strains used in this study were on enriched C57BL/6 background.

### Cell Sorting by Flow Cytometry

Cells were isolated from hindlimb [limb; pooled tibialis anterior (TA), extensor digitorum longus (EDL) and gastrocnemius] or diaphragm muscles of floxed FGFR and control mice harboring the MyoD^Cre^ and the R26^mTmG^ alleles. The R26^mTmG^ reporter operates on a membrane-localized dual fluorescent system where all cells express Tomato until Cre-mediated excision of the Tomato gene allows for GFP expression in the targeted cell lineage (Muzumdar et al., 2007). Consequently, when the R26^mTmG^ allele is combined with MyoD^Cre^ allele all skeletal muscles and their resident SCs are GFP^+^ (Stuelsatz et al., 2014) due to ancestral MyoD expression in the myogenic lineage (Kanisicak et al., 2009). Using this muscle-specific reporter model, the isolated cells are sorted into myogenic and non-myogenic populations according to GFP vs. Tomato fluorochrome, respectively, combined with antigen-based sorting for maximal purification as we previously described (Stuelsatz et al., 2014). In brief, cell suspensions were released from harvested muscles by collagenase/dispase digestion and were first incubated with 10 μM Hoechst 33342 (Sigma-Aldrich) for 30 min at 37°C to label cell nuclei, followed by incubation with the following fluorescently conjugated antibodies (from eBioscience): anti-Sca1 (APC, clone D7), anti-CD31 (PECy7, clone 390), anti-CD45 (PECy7, clone 30-F11). Cell sorting was then performed using an Influx Cell Sorter (BD Biosciences) equipped with 350, 488, and 638 nm lasers. All sorted cells were collected within the G0-G1 population depleted of CD31^+^ (endothelial) and CD45^+^ (hematopoietic) cells, with myogenic and non-myogenic populations isolated as GFP^+^/Sca1^-^ and Tomato^+^/Sca1^+^ cells, respectively. Gates were determined by comparing fluorophore signal intensities between the unstained control and each single antibody/fluorophore control. Data was acquired at 20,000–100,000 events per sample and sorted cells were collected in our culture media described below. Subsequent analysis and flow cytometry plots were generated using FlowJo (TreeStar). Sorted populations were either used as freshly isolated cells for gene expression studies or first expanded in primary cultures before harvested for DNA/RNA isolation and subsequent PCR/RT-PCR analyses as detailed next.

### Primary Cultures of Sorted Myogenic and Non-myogenic Populations

Cells were cultured according to our routine procedures for mouse primary cultures (Danoviz and Yablonka-Reuveni, 2012; Stuelsatz et al., 2014). The basal solution used for all culture medium preparations consisted of Dulbecco’s modified Eagle’s medium (DMEM, high glucose, with L-glutamine, 110 mg/l sodium pyruvate, and pyridoxine hydrochloride, Hyclone) supplemented with antibiotics (50 U/ml penicillin and 50 mg/ml streptomycin, Gibco-Life Technologies). Sorted cells were cultured in 12-well culture plates pre-coated with Matrigel (BD Biosciences, diluted to a final concentration 1 mg/ml) using our standard DMEM-based medium containing 20% fetal bovine serum (Gibco-Life Technologies), 10% horse serum (Gibco-Life Technologies), and 1% chicken embryo extract [prepared from whole 10-day-old embryos as detailed in Notes #4 and 5 in (Danoviz and Yablonka-Reuveni, 2012)] and were incubated at 37°C, 5% CO_2_. Cultures were initiated at a density of 1–2 × 10^4^ cells per well. After the initial plating, growth medium was replaced every 3 days.

### Quantitative Gene Expression Analysis of Freshly Sorted Cells

RNA was isolated from freshly sorted myogenic and non-myogenic populations and reverse transcribed according to our published procedure (Day et al., 2010). Sorted cell populations were pelleted (400 × g for 10 min followed by 90 s at 12,000 × g) and suspended in the lysis buffer from the RNeasy Plus Micro kit (Qiagen) used to isolate total RNA. The RNA was then quantified using an Agilent Bioanalyzer and reverse transcribed (at 0.4 ng/μl) into cDNA using the iScript reverse transcriptase (Bio Rad). Gene expression was determined by SYBR Green-based quantitative PCR using 1 μl cDNA per reaction (20 μl final volume) on an ABI 7300 Real Time PCR machine (Life Technologies) as we previously described (Phelps et al., 2013) except that the annealing temperature for Fgfr1 and Fgfr2 primer sets was adjusted at 66°C instead of the standard 63°C used for the remaining primer sets. Raw qPCR cycle threshold values for each individual sample were normalized to eukaryotic translation elongation factor 2 (*Eef2*) reference gene expression as in (Phelps et al., 2013). Each sample was analyzed in triplicate. Genes were considered expressed if cycle threshold values (raw Ct) of less than 33 cycles were detected.

Primer sequences were (fwd/rev): *Pax7*, GCCACAGCTTCTCCAGCTAC/CACTCGGGTTGCTAAGGATG (120 bp, UCSC Genome Browser ID Pax7_uc008vms.1_1_1_2); *Fgfr1*, GCCCTGGAAGAGAGACCAGC/GAACCCCAGAGTTCATGGATGC [244 bp, (Kwiatkowski et al., 2008)]; *Fgfr2*, GCCTCTCGAACAGTATTCTCCT/ACAGGGTTCATAAGGCATGGG [103 bp, PrimerBank ID 2769639a1, (Spandidos et al., 2010)]; *Fgfr3*, GGCTCCTTATTGGACTCGC/TCGGAGGGTACCACACTTTC [219 bp, (Deng et al., 1996)]; *Fgfr4*, TTGGCCCTGTTGAGCATCTTT/GCCCTCTTTGTACCAGTGACG (189 bp, PrimerBank ID 6679789a1); *Eef2*, TGTCAGTCATCGCCCATGTG/CATCCTTGCGAGTGTCAGTGA (123 bp, PrimerBank ID 33859482a1). The final concentration of all primers was 500 nM.

### Genomic and Transcriptional Analysis of Cultured Cells

Sorted cells cultured for 7 days were rinsed twice with DMEM before adding the lysis buffer from the AllPrep DNA/RNA Mini kit (Qiagen) used for simultaneous purification of genomic DNA and total RNA. Resulting preparations were quantified with a NanoDrop spectrophotometer. Genomic analyses were done by using 5 μl of DNA solution (adjusted to 10 ng/μl) per PCR reaction (25 μl final volume). PCR primers used for Fgfr1 and Fgfr2 genomic products (wildtype, flox and Δ) were according to (White et al., 2007). Transcript expression analysis was done by semi-quantitative RT-PCR according to our standard protocol (Day et al., 2007). Briefly, the RNA was reverse transcribed (at 20 ng/μl) into cDNA using the iScript reverse transcriptase (Bio Rad) and 5 μl of cDNA per PCR reaction (25 μl final volume) were used. PCR primers used for transcript expression analysis were previously described by us in (Kwiatkowski et al., 2008; Stuelsatz et al., 2012) and were used here at a final concentration of 400 nM. Expression of Tbp (TATA box binding protein) housekeeping control gene served as quality and loading control as in (Stuelsatz et al., 2012). For all PCR reactions, the following cycling parameters: 95°C for 15 min, 22–30 cycles of 94°C for 40 s, 60°C for 50 s, 72° for 1 min, with a final extension step of 72°C for 10 min were used. PCR products were separated on 1.5% agarose gels containing 1:10,000 dilution of SYBR Green I (Molecular Probes). Gels were imaged using Gel Logic 212 Pro (Carestream).

### Quantification of SCs on Isolated Myofibers

Single myofibers were isolated from the EDL muscle as we previously described (Day et al., 2010; Keire et al., 2013). For each mouse strain and for each condition tested, myofibers were typically isolated from 3 mice. For analyzing the number of SCs on freshly isolated myofibers, we relied on Pax7 immunostaining following our standard approach using adherent myofibers where each myofiber is dispensed into an individual Matrigel-coated well (Shefer et al., 2006; Day et al., 2007; Keire et al., 2013) prior to fixation and immunostaining. For analyzing SC proliferation/differentiation, myofibers were cultured for 3 days in non-coated wells (24-well trays, 1 myofiber per well) using a DMEM-based medium containing 10% horse serum, an approach that yields non-adhering myofibers and maintains the SCs and their progeny associated with the parent myofibers [adapted from (Zammit et al., 2004)]. For myofibers treated with FGF, FGF2 was supplemented at 5 ng/ml (R&D Systems, recombinant human FGF basic, #234-FSE-025). The cultures were initiated in 0.3 ml and the replenishment of the medium (±FGF2) was achieved by adding fresh medium (0.2 ml) on culture day 1 and performing partial medium change (0.25 ml) on culture day 2; this approach ensured that myofibers were not disturbed during medium change. Myofibers were fixed on day 3 by adding to the medium an equal volume of 4% paraformaldehyde [PFA, prepared as detailed in Note# 14 in (Keire et al., 2013)]. SCs were analyzed by immunostaining using mouse antibodies against Pax7 [Developmental Studies Hybridoma Bank (DSHB), ascites, 1:1000], MyoD (BD Biosciences, 1:800), Myogenin (DSHB, supernatant, 1:5) and counterstaining with DAPI according to our standard protocol for blocking, rinsing and mounting the myofibers (Shefer et al., 2006; Keire et al., 2013), except that extra care had to be taken due to the non-adherent nature of the myofibers.

### Muscle Injury and Histology

Mice were anesthetized with isoflurane. For each mouse, the TA muscle from one leg was injected with 25 μl of 20 μM cardiotoxin (Sigma C9759), while the TA from the contralateral leg was injected with 25 μl of 0.9% NaCl as a control. TAs (with EDLs attached, referred later as TA/EDL) were harvested at different time points after injury, embedded in OCT (Tissue-Tek) and flash frozen in isopentane cooled with liquid nitrogen. Transverse sections (10 μm) prepared using a Leica CM1850 cryostat were stained with hematoxylin and eosin [H&E, as described in (Stuelsatz et al., 2015)] or alternatively fixed with 2% PFA for 10 min before being stained with DAPI when analyzed for GFP and Tomato fluorochrome expression.

### FGFR4 Immunodetection

FGFR4 immunolabeling was performed on unfixed cryosections or on fixed primary myogenic cultures processed according to our standard protocol (Kwiatkowski et al., 2008; Stuelsatz et al., 2014). Cultures were prepared from Pronase digested muscle and grown on gelatin as in (Danoviz and Yablonka-Reuveni, 2012) before being fixed with ice-cold methanol as we previously published (Yablonka-Reuveni and Rivera, 1997a). In all cases, specimens were prepared from limb muscle of wildtype mice. Rabbit anti-FGFR4 was either from Santa Cruz Biotechnology or produced in our laboratory [(Kwiatkowski et al., 2008), available from Millipore]. FGFR4 immunolabeling of cryosections was done in combination with laminin immunodetection (Stuelsatz et al., 2014) to identify presumptive SCs based on their location underneath the myofiber basal lamina.

### Microscopy and Imaging

Cell culture and histological observations were made with an inverted fluorescent microscope (Eclipse TE2000-S, Nikon). Images were acquired using CoolSNAP ES monochrome CCD camera (Photometrics) controlled with MetaVue Imaging System (Universal Imaging Corporation). For acquiring real color images of H&E stained muscle sections, images were taken with a Digital Sight DS-Ri1 color camera controlled by NIS-Elements F software (Nikon). Digitized images were assembled using Adobe Photoshop software. For final images of tissue cross sections showing the whole TA/EDL muscle, several pictures were taken (with a 10 or 20x objective) and merged together, resulting in a high-resolution view of the entire muscle cross-sectional area.

### Statistics

Data were analyzed by one-way ANOVA (*p* < 0.05) with Bonferroni–Holm *post hoc* analysis using Excel with Daniel’s XL Toolbox Add-In (by Daniel Kraus, Würzburg, Germany).

## Results and Discussion

### Experimental Approach

**FIGURE 1:**
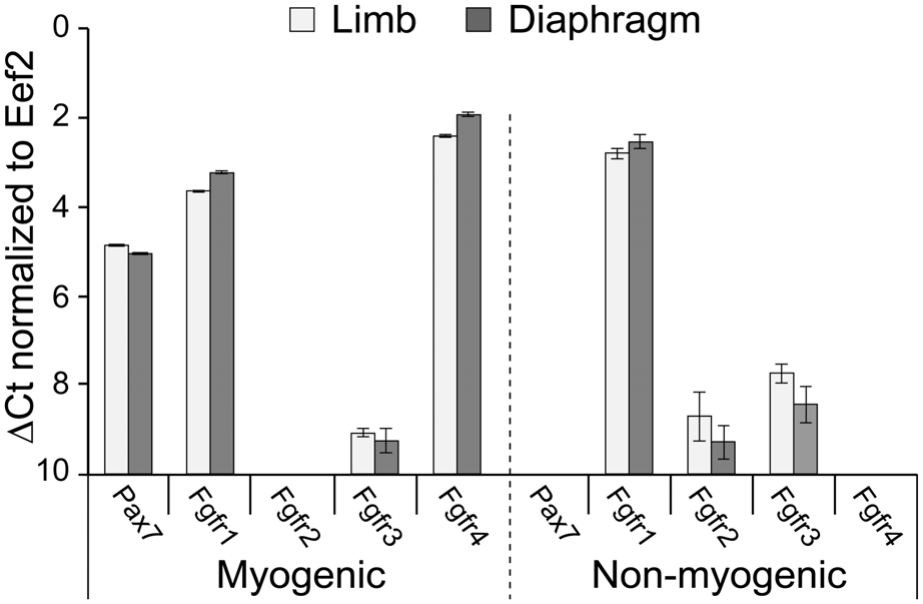
**Fgfr expression in freshly isolated SCs from limb and diaphragm muscles of MyoD^Cre/+^/R26^mTmG/+^ mice.** Myogenic and non-myogenic cell populations were sorted by flow cytometry (based on GFP and Tomato fluorescence, respectively, and cell surface antigens) and analyzed by quantitative RT-PCR. Gene expression values were normalized to *Eef2* reference gene expression (ΔCt). Average Ct values for Eef2 gene (±SD) were 23.49 ± 0.09 (limb myogenic), 22.90 ± 0.04 (diaphragm myogenic), 20.84 ± 0.01 (limb non-myogenic), and 20.42 ± 0.01 (diaphragm non-myogenic).

**FIGURE 2:**
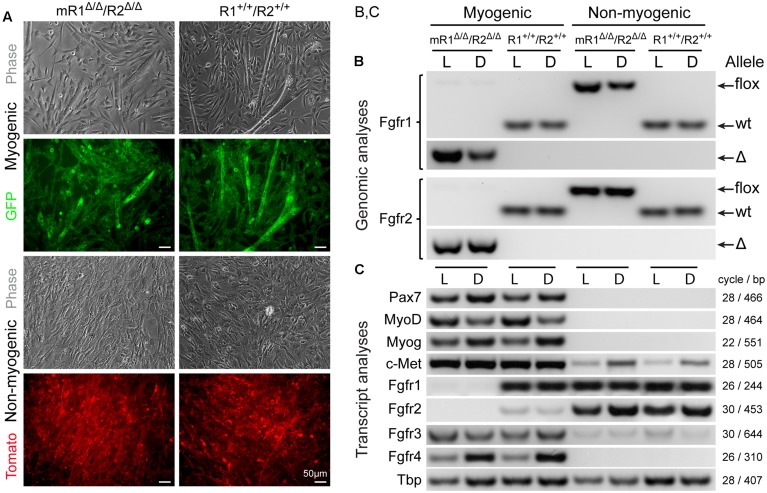
**MyoD-driven Cre induces effective deletions of Fgfr1 and Fgfr2 in the myogenic lineage without modulating gene expression levels of Fgfr3 and Fgfr4.** Myogenic (GFP^+^) and non-myogenic (Tomato^+^) cell populations were sorted by flow cytometry (as in **Figure 1**) from limb and diaphragm muscles (denoted as L and D, respectively) of MyoD^Cre/+^/R26^mTmG/+^/Fgfr1^fl/fl^/Fgfr2^fl/fl^ (mR1^Δ/Δ^/R2^Δ/Δ^) and control MyoD^Cre/+^/R26^mTmG/+^ (R1^+/+^/R2^+/+^) mice. **(A)** Representative images of sorted GFP^+^ and Tomato^+^ cell populations isolated from hindlimb muscles and cultured for 7 days before being processed for simultaneous DNA and RNA isolation and further PCR and RT-PCR analyses, respectively. As shown here, the myogenic (GFP^+^) cultures displayed the initiation of myotube formation that became more prominent by culture days 10–14 (not shown), while the non-myogenic (Tomato^+^) cultures were void of myotubes. **(B)** PCR analysis of the presence of the different Fgfr1 and Fgfr2 alleles (wt, flox, or Δ alleles) at the genomic level. The detection of PCR products of the Cre-mediated genomic deletions (Δ) solely in myogenic (GFP^+^) cells confirms the muscle-specific deletion of Fgfr1 and Fgfr2 genes. **(C)** Semi-quantitative RT-PCR analysis of Fgfr transcript levels. Fgfr1 and Fgfr2 transcripts were absent in myogenic (GFP^+^) cells from mR1^Δ/Δ^/R2^Δ/Δ^ mice (in agreement with the genomic analysis), while expressed at a relatively high (Fgfr1) and low (Fgfr2) levels, in control myogenic (GFP^+^) cells from R1^+/+^/R2^+/+^ mice. In contrast, Fgfr3, Fgfr4, and c-Met were each detected at a similar level in the myogenic cultures from mR1^Δ/Δ^/R2^Δ/Δ^ vs. R1^+/+^/R2^+/+^ mouse strains. The observed higher Fgfr4 expression levels in diaphragm (vs. limb) myogenic cultures from both mR1^Δ/Δ^/R2^Δ/Δ^ and R1^+/+^/R2^+/+^ mice appear to coincide with the higher myogenin expression levels observed.

To achieve muscle-specific ablation of FGFR1 we have used a Cre/loxP genetic approach relying on the MyoD^Cre^ allele to mediate ablation of the floxed Fgfr1 gene. Regardless of muscle origin, virtually all SCs in adult muscles are derived from progenitors that have expressed the MyoD^Cre^ allele during embryogenesis (Kanisicak et al., 2009; Yamamoto et al., 2009). Hence, as detailed in the Introduction, MyoD^Cre^-mediated excision of floxed genes would occur in the embryonic muscle and be stably maintained in the myogenic lineage through adult life. Indeed, our use of the R26^mTmG^ mouse (a floxed dual fluorescent reporter system described in Materials and Methods), crossed with the MyoD^Cre^ mouse, has clearly demonstrated specificity of the MyoD^Cre^-mediated excision (i.e., GFP expression) in all adult muscles in both myofibers (which are formed during embryogenesis by myoblasts fusion) and SCs (Stuelsatz et al., 2012, 2014, 2015). Moreover, this specific expression of GFP in the myogenic lineage of MyoD^Cre^ × R26^mTmG^ mice has provided us with an effective tool for sorting SCs (GFP^+^) from non-myogenic (Tomato^+^) populations (Stuelsatz et al., 2012, 2014, 2015). While we were mostly interested in the present study in the role of FGFR1, the founder mice we had received to establish our colony harbored both floxed Fgfr1 and Fgfr2 alleles. As detailed in the Introduction, Fgfr2 has been considered not relevant in adult myogenesis and indeed, as shown in **Figures 1** and **2**, its expression level in SCs and their progeny is negligible. However, Fgfr2 could have theoretically been upregulated in the cell culture conditions used in the current study and/or upon Fgfr1 ablation. Hence, in this original investigation of the effect of Fgfr genetic ablation on the myogenic lineage we decided to retain both Fgfr1 and Fgfr2 floxed alleles. Mice carrying these myogenic-specific (MyoD^Cre^-driven) double homozygous deletions are referred to throughout the manuscript as mR1^Δ/Δ^/R2^Δ/Δ^, while control mice, wildtype for Fgfr1 and Fgfr2, or harboring floxed Fgfr1 and Fgfr2 alleles, are referred to as R1^+/+^/R2^+/+^ or R1^fl/fl^/R2^fl/fl^, respectively. The mR1^Δ/Δ^/ R2^Δ/Δ^ mice (with or without the R26^mTmG^ allele) were fertile and appeared normal by size and overall morphology (mice were followed up to 16 months of age).

### Fgfr Expression in Freshly Isolated SCs

Before embarking on Fgfr ablation, we wished to analyze endogenous Fgfr transcript levels in freshly isolated SCs in comparison with non-myogenic cells. Gene expression analyses were performed on freshly isolated populations sorted from limb and diaphragm muscles of MyoD^Cre/+^/R26^mTmG/+^ mice (**Figure 1**, quantitative RT-PCR). For both muscle types analyzed, the Pax7 data validates the myogenic nature of the GFP^+^ population; i.e., Pax7, the classic marker of SCs, was expressed only by the sorted GFP^+^ population but not by the Tomato^+^ non-myogenic population (**Figure 1**). As additionally shown in **Figure 1**, Fgfr1 was expressed at a relatively high level by both the myogenic and non-myogenic populations, while Fgfr4 was expressed only by the myogenic population, in accordance with our previous rat studies (Kastner et al., 2000). Fgfr2 was below detection level in the myogenic population, while some Fgfr2 expression was demonstrated by the non-myogenic population. Fgfr3 was detected at relatively low level in both the myogenic and non-myogenic populations (**Figure 1**).

### MyoD^Cre^ Induces Effective Deletions of the Floxed Fgfr1 and Fgfr2 Alleles in the Myogenic Lineage without Modulating Endogenous Levels of Fgfr3 and Fgfr4

The efficiency of MyoD^Cre^-driven Fgfr1/Fgfr2 deletions in SCs was evaluated concurrently at the genomic (PCR) and transcript (RT-PCR) levels for both limb and diaphragm muscles (**Figure 2**). The cells were isolated from mR1^Δ/Δ^/R2^Δ/Δ^ and control R1^+/+^/R2^+/+^ mice that also harbored the R26^mTmG^ reporter to facilitate cell sorting of SCs vs. non-myogenic cells and to confirm the purity of the sorted populations in culture according to GFP vs. Tomato reporter color, respectively (**Figure 2A**). To ensure sufficient material for the analyses, and also to obtain insight into possible modulations in Fgfr gene expression upon proliferation/differentiation vs. freshly isolated cells (**Figure 1**), the sorted cells were cultured for 7 days in our standard rich–medium conditions, then harvested for simultaneous isolation of DNA and RNA preparations.

Notably, there were no apparent differences in overall morphology of the myogenic cultures from Fgfr1/Fgfr2-ablated (mR1^Δ/Δ^/R2^Δ/Δ^) and control (R1^+/+^/R2^+/+^) mice, whether cells were isolated from limb (**Figure 2A**) or diaphragm muscles (data not shown). For both mouse strains, the cultured GFP^+^ cells demonstrated typical myogenic features, fusing into myotubes by day 7 (**Figure 2A**), with myotubes enlarging in number and size in subsequent days (not shown). The non-myogenic cultures (Tomato^+^) from both Fgfr-deleted and control mice harbored typical features of fibroblastic cells as expected, with no myotubes detected even when following the cultures for longer time.

The genomic analysis of the different Fgfr1 and Fgfr2 alleles (wt, flox, or Δ alleles) validated that the mice harbored the anticipated alleles in accordance with mouse genotype and cell type analyzed (**Figure 2B**). The detection of genomic PCR products specific of the MyoD^Cre^-mediated Fgfr1 and Fgfr2 genomic deletions (Δ allele) solely in myogenic cells confirmed muscle-specific deletions while the concurrent absence of any residual flox allele revealed the high efficiency of the Cre-mediated recombination in the SC lineage.

Fgfr transcript evaluation by semi-quantitative RT-PCR in cultures from both limb (L) and diaphragm (D) further demonstrates the effectiveness of MyoD^Cre^-driven Fgfr1-ablation in the myogenic lineage (**Figure 2C**) while Fgfr2 is already barely detected in the myogenic lineage from the non-ablated control. One primer of each pair used to detect Fgfr1 or Fgfr2 transcripts is localized within the targeted floxed region, thereby avoiding detection of truncated mRNAs that may be produced by the Δ alleles. Nevertheless, mutant FGFR proteins potentially translated from such truncated mRNA would be non-functional due to the lack of critical domains (see Materials and Methods). Indeed, as anticipated based on their location within the corresponding Fgfr floxed region, our Fgfr1/Fgfr2 primers did not produce any RT-PCR products when analyzing Fgfr1/Fgfr2 mRNA expression in the myogenic lineage from mR1^Δ/Δ^/R2^Δ/Δ^ mice (**Figure 2C**). This is in contrast to that seen in non-myogenic cell cultures where both Fgfr1 and Fgfr2 are expressed at a relatively high level for both mouse strains analyzed (**Figure 2C**), demonstrating the specificity of the Fgfr1/Fgfr2 ablation to the myogenic lineage. Fgfr3 and Fgfr4 expression levels in myogenic cells were unaffected when comparing myogenic cells from mR1^Δ/Δ^/R2^Δ/Δ^ vs. R1^+/+^/R2^+/+^ muscles. Likewise, the level of c-Met, the receptor for HGF, also an established mitogen of SCs as detailed in the Introduction, was unaffected following Fgfr1/Fgfr2 deletion (**Figure 2C**). Hence, there is no apparent compensatory upregulation of Fgfr3, Fgfr4, or c-met in the Fgfr1/Fgfr2-ablated myogenic lineage.

The data in **Figure 2C** illustrate additional noteworthy points regarding Fgfr expression in cultures from both limb (L) and diaphragm (D) in the context of the control R1^+/+^/R2^+/+^ cultures. (i) Fgfr3 appears to be expressed at a higher expression level in the myogenic cultures vs. the non-myogenic cultures and Fgfr4 is clearly expressed only in the myogenic cultures. (ii) When compared to Fgfr expression levels in freshly isolated populations from R1^+/+^/R2^+/+^ control mice (**Figure 1**), Fgfr1 and Fgfr4 appear to retain the same expression profile in the day 7 cultures (with no Fgfr4 being detected in the non-myogenic cells), but Fgfr2 and Fgfr3 appear to be up-regulated in the cultured non-myogenic and myogenic cells, respectively. Our additional unpublished studies of limb-derived sorted populations have shown that Fgfr2 expression level continues to rise in the non-myogenic population with time in culture, concomitant with adipogenic differentiation that takes place uniquely in this Sca1^+^ sorted population. The latter non-myogenic population has previously been defined by others and us as fibro/adipogenic progenitors (Joe et al., 2010; Stuelsatz et al., 2014).

### Muscle Tissue of Adult mR1^Δ/Δ^/R2^Δ/Δ^ Mice Does Not Show Apparent Signs of Histopathology or Abolishment of Regenerative Activity

**FIGURE 3:**
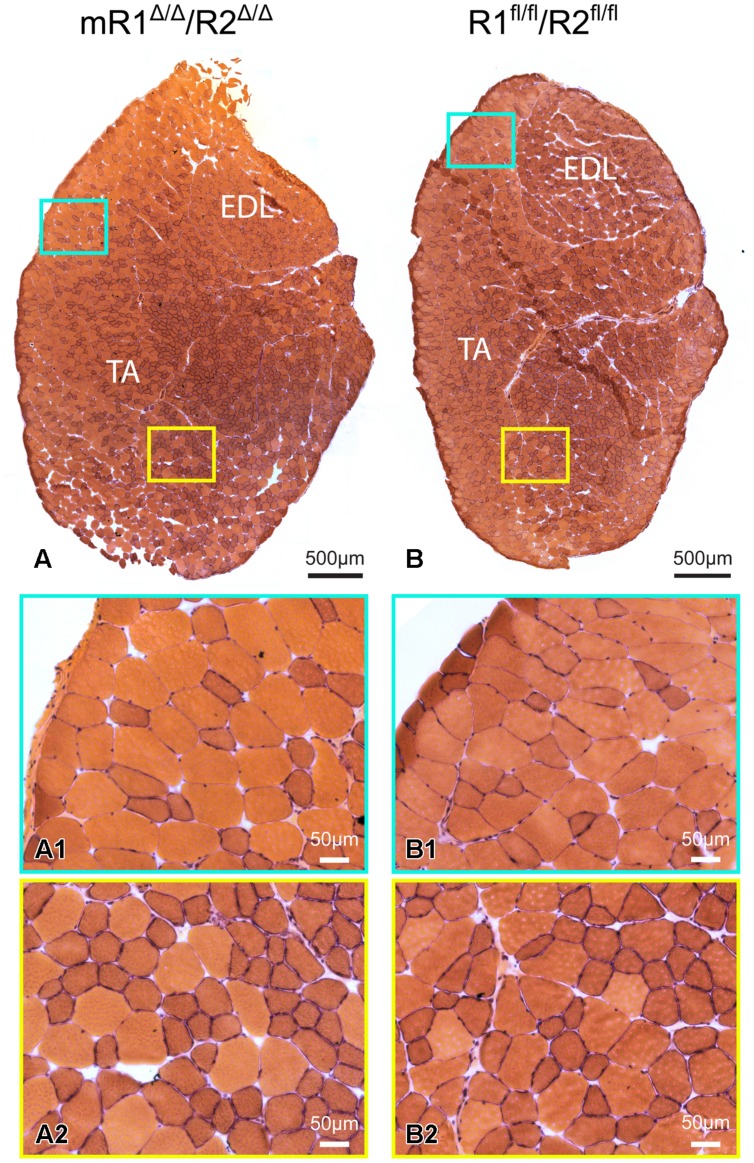
**Muscle tissue of adult mR1^Δ/Δ^/R2^Δ/Δ^ mice does not appear different from that of control muscle from R1^+/+^/R2^+/+^ mice.** Representative images of H&E stained cross sections of TA/EDL from 10-month-old **(A)** mR1^Δ/Δ^/R2^Δ/Δ^ and **(B)** R1^+/+^/R2^+/+^ mice. For each panel, regions delineated in the low magnification image of the whole TA/EDL **(A,B)** are shown as higher magnification views **(A1–B2)** identified with corresponding colored frames. Muscles from both mouse strains harbored typical histology with larger and smaller diameter myofibers with peripheral nuclei.

**FIGURE 4:**
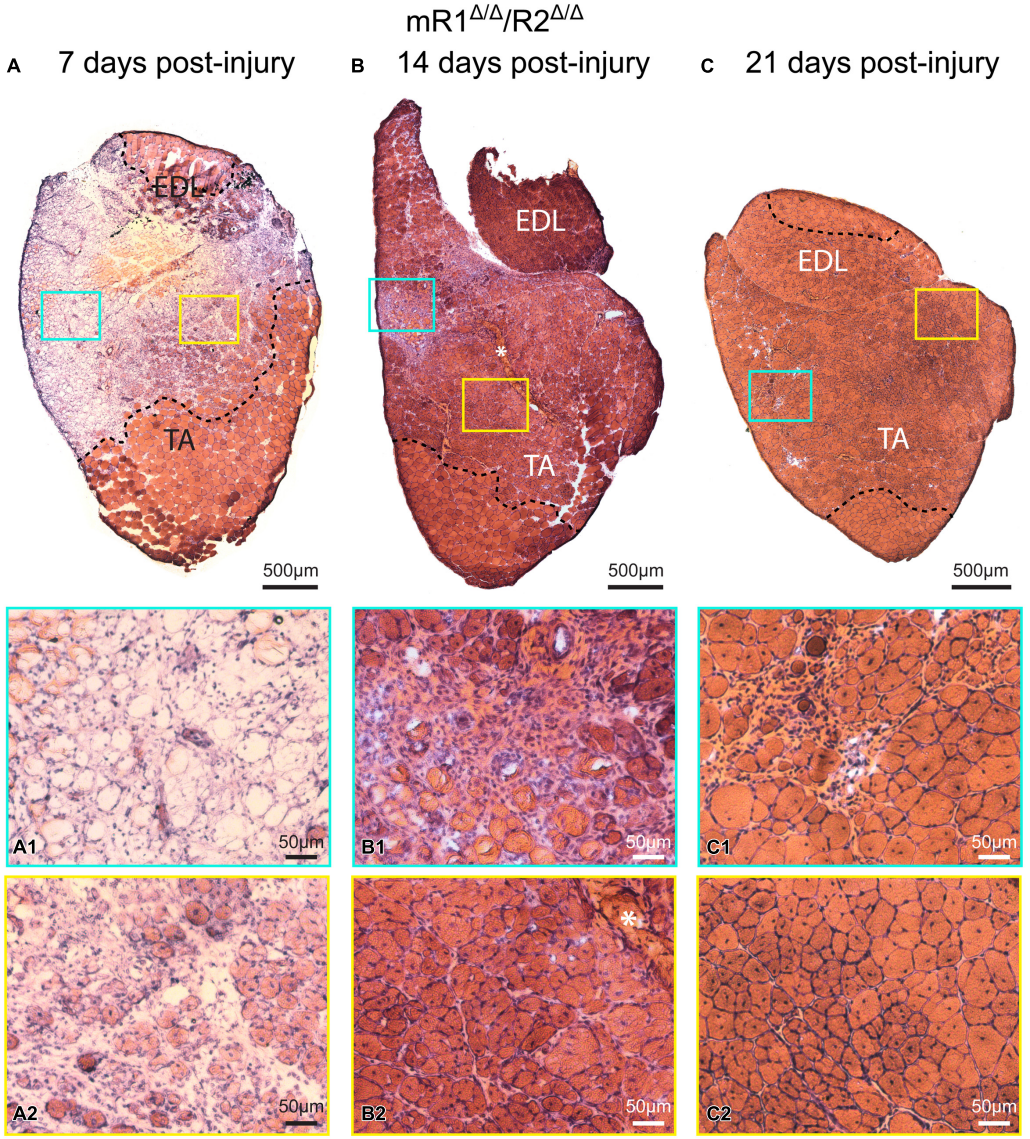
**Muscle tissue of adult mR1^Δ/Δ^/R2^Δ/Δ^ mice retains regenerative activity.** Representative images of H&E stained cross sections of TA/EDL from 4-month-old mR1^Δ/Δ^/R2^Δ/Δ^ mice, showing extensive damage at 7 days post cardiotoxin-induced injury, and progressive recovery at 14 and 21 days post-injury. For each panel, regions delineated in the low magnification image of the whole TA/EDL are shown as higher magnification views **(A1–C2)** identified with corresponding colored frames; dotted lines in the low magnification images delineate the outer limits of the region that has been effectively injured. Morphology of control contralateral TAs (NaCl-injected, not shown) appeared similar to that of the uninjured muscle depicted in **Figure 3**. **(A)** As seen on day 7 post-injury, cardiotoxin injection caused massive myofiber degeneration, resulting in large necrotic regions in which empty remnants of the original myofibers **(A1)** and infiltration of inflammatory cells **(A2)** are detected; regions with small regenerating myofibers with central myonuclei (hallmark of regenerating myofibers) were occasionally observed **(A2)**. **(B)** On day 14 post-injury, regenerating myofibers were more abundant **(B2)**, but regions showing infiltration of inflammatory cells were still occasionally present **(B1)**; asterisk in **(B)** and **(B2)** indicates the scar left at the needle injection point. **(C)** By day 21 post-injury, most of the original injured region showed successful regeneration based on the presence of larger (relative to day 14) myofibers containing central nuclei and overall tissue morphology **(C2)**; infiltration of inflammatory cells was only minimally detected at this stage **(C1)**.

**FIGURE 5:**
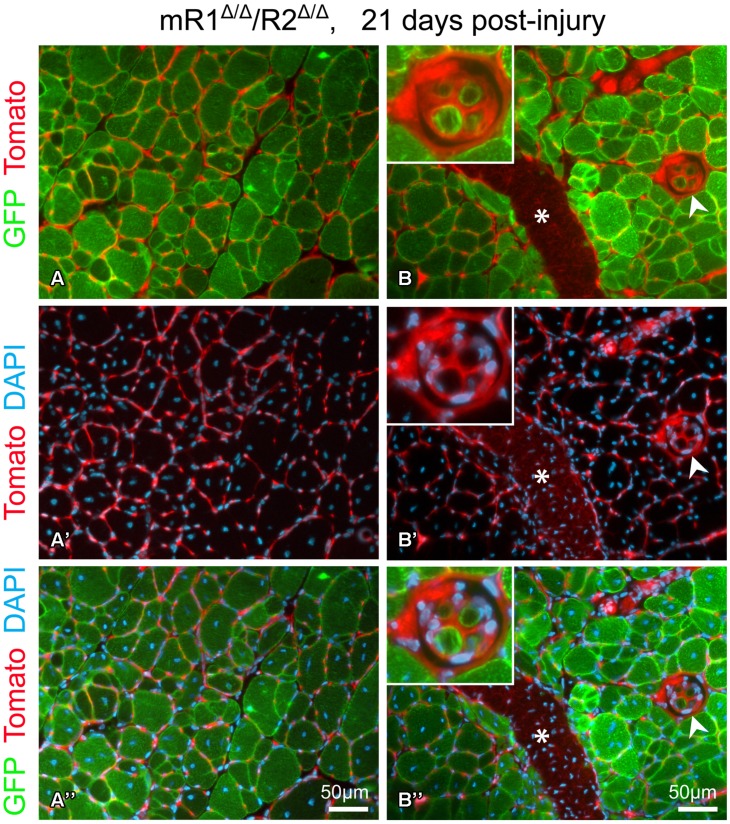
**Fluorescent images of cross sections prepared from TA isolated 21 days post-injury from a 4-month-old mR1^Δ/Δ^/R2^Δ/Δ^ mouse (also harboring the R26^mTmG^ allele) depicting GFP and Tomato fluorescence, indicative of myogenic and non-myogenic structures, respectively, with DAPI^+^ nuclei. (A–A”)** The use of the R26^mTmG^ allele together with the MyoD^Cre^ driver (used for recombining the floxed Fgfr1 and Fgfr2 alleles) demonstrates that as expected, the regenerated myofibers identified by their central nuclei, were GFP^+^, hence, of MyoD lineage origin. The capillaries and connective tissue surrounding myofibers are Tomato^+^ (i.e., of non-MyoD^+^ origin). **(B–B”)** In addition to the standard myofibers (extrafusal), a muscle spindle (arrowhead, higher magnification view in top left insert) can be observed within a regenerating region. While the spindle capsule and the material surrounding each intrafusal myofiber are of a non-MyoD^+^ origin (Tomato^+^), similar to the standard myofibers, the intrafusal myofibers are of MyoD-lineage origin (GFP^+^). Note the distinctive smaller diameter size of the intrafusal myofibers compared to the larger extrafusal myofibers. Asterisk indicates the scar (Tomato^+^) left at the needle injection point. Notably, as shown in panels **(A)** and **(B)**, sites with groups of smaller diameter extrafusal myofibers were observed in addition to the larger diameter myofibers. Morphology of control contralateral TAs (NaCl-injected, not shown) exhibited no differences when compared to uninjured muscle depicted in **Figure 3**.

Histological examination of muscle tissues from Fgfr1/Fgfr2-ablated mice showed no apparent differences compared to the control (R1^fl/fl^/R2^fl/fl^) mice. Low and high magnification images of H&E stained cross sections processed from TA/EDL of mR1^Δ/Δ^/R2^Δ/Δ^ and control R1^fl/fl^/R2^fl/fl^ mice demonstrate for both mouse strains a normal muscle morphology (**Figure 3**). Next, we analyzed muscle regeneration in mR1^Δ/Δ^/R2^Δ/Δ^ mice (**Figures 4** and **5**) following intramuscular administration of cardiotoxin, which specifically destroys the myofibers but preserves SCs (Harris, 2003). As seen in **Figure 4**, while most of the cardiotoxin-injected muscle tissue did not initiate myofiber formation on day 7 post-injury and still demonstrated large areas of inflammatory cell infiltrations at day 14, by day 21 there was an effective regenerative process throughout the muscle as observed by the characteristic presence of central myonuclei (**Figure 4**). Our unpublished studies with wildtype adult mice have demonstrated formation of nascent regenerative myofibers by day 7 following cardiotoxin injury and an almost complete myofiber recovery by day 14 post-injury. Hence, it appears that mR1^Δ/Δ^/R2^Δ/Δ^ injured muscle has a lag in muscle regeneration. Nevertheless, our data (**Figures 4** and **5**) clearly indicate a thorough regeneration of the injured muscle by day 21 regardless of Fgfr1/Fgfr2 ablation in the myogenic lineage.

This injury study presented in **Figures 4** and **5** was done in mR1^Δ/Δ^/R2^Δ/Δ^ mice that also harbored the R26^mTmG^ allele to facilitate direct tracking of myogenic cells/myofibers (GFP^+^) vs. non-myogenic cells (Tomato^+^), and as expected the newly regenerated myofibers are of MyoD lineage origin (**Figure 5**). The GFP reporter has also permitted the observation of (i) infrequent groups of small-diameter myofibers (**Figure 5A**), and (ii) the tiny intrafusal myofibers (**Figure 5B**) constituting the muscle spindle apparatus that plays a role in proprioception (Walro and Kucera, 1999; Kirkpatrick et al., 2008). Interestingly, the muscle spindle seen in **Figure 5B** is located within a regenerating region characterized by central myonuclei and thus most likely underwent a regeneration process similar to the surrounding myofibers.

### SCs in Isolated Myofibers From mR1^Δ/Δ^/R2^Δ/Δ^ Mice Exhibit Impaired Proliferative Response to FGF2

**FIGURE 6:**
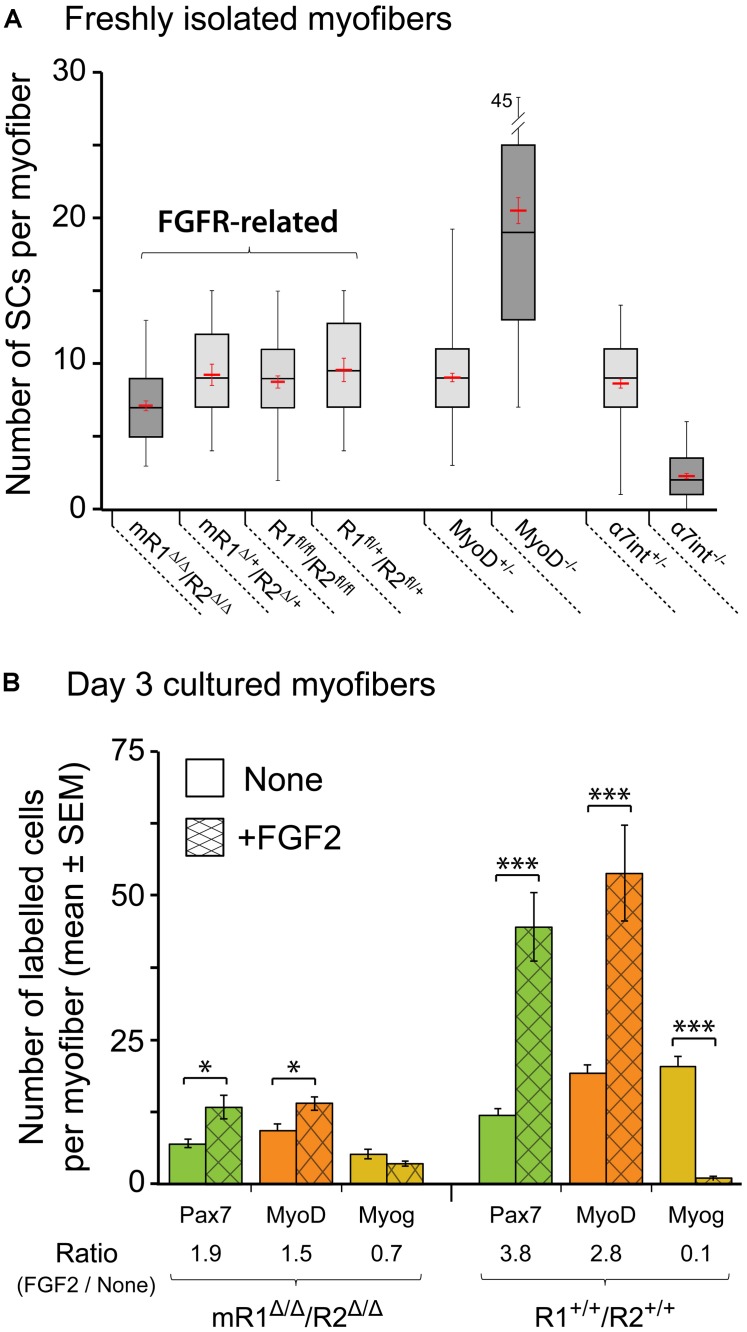
**Satellite cells (SCs) in isolated EDL myofibers from mR1^Δ/Δ^/R2^Δ/Δ^ mice do not display a drastic change in their number but exhibit impaired proliferative response to FGF2. (A)** Quantification of SCs in freshly isolated myofibers from different mouse strains as listed under the *X*-axis. SCs were quantified on individual myofibers by Pax7 immunostaining combined with DAPI-staining to highlight both SCs and myonuclei. Data are summarized as boxplots, depicting the quartile distribution and mean ± SEM (red marks) for the number of SCs per myofiber; the whiskers on each side of the box are taken to the minimum and maximum values. MyoD-null and α7integrin-null data are included for comparison, as these mutations do drastically affect SC numbers. For each strain as listed from left to right under the *X*-axis, the number of myofibers analyzed was 48, 18, 54, 18, 120, 96, 88, and 95, respectively. **(B)** Single myofibers were maintained in suspension for 3 days with or without FGF2 supplement (5 ng/ml), then fixed and analyzed by immunostaining for the expression of the myogenic markers Pax7, MyoD and myogenin as a means to investigate SC dynamics. For typical Pax7/MyoD/myogenin immunostaining images see our previous mouse myofiber studies (Yablonka-Reuveni et al., 1999a; Shefer et al., 2006; Keire et al., 2013); examples of MyoD staining that depict the proliferative response of SCs to FGF2 supplementation are shown in **Figure 7**. To quantify the effect of FGF2 on SCs, the ratio in average cell numbers between FGF2-treated and untreated myofibers was determined for each marker (indicated under *X*-axis legend). Asterisks denote statistically significant differences in the number of labeled cells per myofiber between FGF2-treated and untreated myofibers (single asterisk *p* < 0.05; triple asterisks *p* < 0.001). For each condition as listed from left to right under the *X*-axis, the number of myofibers analyzed was 19, 21, 18, 21, 16, 17, 17, 12, 16, 12, 15, and 13, respectively.

Based on the outcome of the injury study described above, FGFR1/FGFR2 do not appear to be essential (at least at the histological level) for muscle regeneration following cardiotoxin injury, but it does not necessarily preclude a role for FGF signaling system in muscle regeneration. Indeed, multiple growth factors have been implicated in muscle regeneration and might compensate functionally for each other role in the cardiotoxin-induced muscle regeneration model (Charge and Rudnicki, 2004; Shefer and Yablonka-Reuveni, 2008). Hence, to directly investigate the impact of Fgfr ablation on SC number and performance, we analyzed isolated myofibers maintained in culture conditions where SCs are retained at their native position by the myofiber as the cells undergo proliferation and differentiation (Yablonka-Reuveni and Rivera, 1994; Zammit et al., 2004; Keire et al., 2013). In the current study myofibers were isolated from EDL muscles and were either allowed to adhere to Matrigel to determine SC numbers on freshly isolated myofibers according to Pax7 immunostaining (**Figure 6A**), or maintained in suspension to investigate SC dynamics (Pax7/MyoD/myogenin immunostaining) in response to FGF2 over 3 days in culture (**Figure 6B**).

The boxplot analysis of freshly isolated EDL myofibers immunostained for Pax7 (**Figure 6A**) suggests that within the four different groups identified as “FGFR-related,” the mR1^Δ/Δ^/R2^Δ/Δ^ mice potentially harbor less SCs per myofiber. An ANOVA test indeed revealed a statistically significant difference. Nevertheless, SC number in myofibers of mR1^Δ/Δ^/R2^Δ/Δ^mice does not appear to be overtly affected when each of the FGFR-related groups are compared with mice lacking MyoD or α7integrin that show a clear increase or decrease, respectively, in their SC numbers (**Figure 6A**). Overall, the number of SCs per myofiber in each of the FGFR-related groups (and in the MyoD^+/-^ and α7integrin^+/-^ groups) all fall within the wildtype range of adult male mice (Shefer et al., 2006; Day et al., 2007, 2010). Notably, the increase in SC numbers in MyoD-null mice was previously recognized (Megeney et al., 1996; Yablonka-Reuveni et al., 1999a; Cornelison et al., 2000; Gayraud-Morel et al., 2007), but while α7integrin has been known to be expressed in the myogenic lineage, including in SCs (Burkin and Kaufman, 1999; Sacco et al., 2008; Rooney et al., 2009; Ieronimakis et al., 2010), we report here the novel finding of significantly reduced SC numbers in the absence of α7integrin.

**FIGURE 7:**
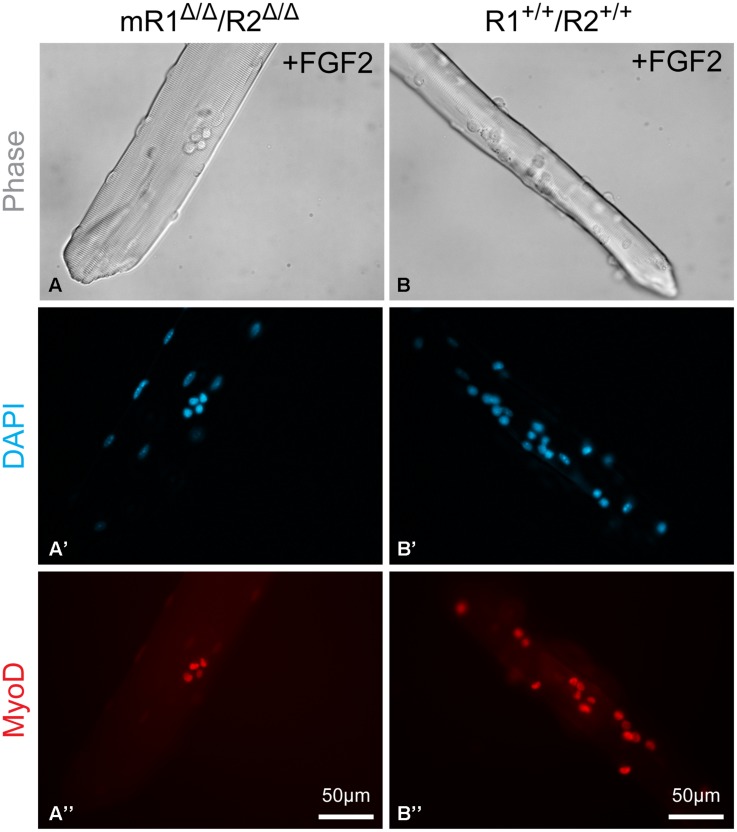
**Examples of EDL myofibers isolated from **(A–A”)** mR1^Δ/Δ^/R2^Δ/Δ^ or **(B–B”)** R1^+/+^/R2^+/+^ mice and cultured in suspension for 3 days with FGF2 supplement and then immunostained for MyoD, which is expressed by proliferating and differentiating SCs.** DAPI counterstaining detected both the MyoD^+^ cells and the myofiber nuclei, but only nuclei at the focal level of the MyoD^+^ cells can be seen in the images shown. The apparent difference in diameter between the two examples of myofibers shown in **(A)** vs. **(B)** is arbitrary and does not reflect a strain difference, as clearly demonstrated by the cross section images shown in **Figure 3**.

To analyze the effect of FGF2 on SC performance, myofibers were maintained for 3 days in suspension in basal medium (DMEM containing 10% horse serum, which is known to contain fewer growth promoting factors than fetal bovine serum) with or without FGF2 supplement. The cultured myofibers were then analyzed by immunostaining using antibodies against Pax7, MyoD and myogenin to quantify SCs and their progeny according to their transcription factor expression status (**Figure 6B**) The FGF2-mediated increase in Pax7^+^ or MyoD^+^ cells seen by day 3 in control (R1^+/+^R2^+/+^) cultures is drastically affected in myofibers from mR1^Δ/Δ^/R2^Δ/Δ^ mice (exemplified by MyoD immunostaining in **Figures 7A–B**”). Indeed, the ratio in average cell numbers between FGF2-treated and untreated myofibers declined by ∼50% in the mR1^Δ/Δ^/R2^Δ/Δ^ mice (1.9 [Pax7] and 1.5 [MyoD]) compared to R1^+/+^/R2^+/+^ mice (3.8 [Pax7] and 2.8 [MyoD] **Figure 6B**). There was a slight decline in Pax7^+^, MyoD^+^, and myogenin^+^ cell numbers in untreated (i.e., not exposed to FGF2) mR1^Δ/Δ^/R2^Δ/Δ^ myofibers. This may be due to the subtle decline in the initial number of SCs noted in freshly isolated myofibers (**Figure 6A**) and/or due to an impaired response of mR1^Δ/Δ^/R2^Δ/Δ^ myofibers to the basal levels of FGF2, available in the cell culture serum or contributed by the myofibers (Yablonka-Reuveni and Rivera, 1994; Chakkalakal et al., 2012). The transition to the differentiated, myogenin^+^ state, was suppressed by FGF2 in the R1^+/+^/R2^+/+^ myofibers (i.e., the ratio of myogenin^+^ cells in FGF2-treated vs. untreated myofibers was 0.1), which is in agreement with the established FGF2 effect on delaying myogenic differentiation (Clegg et al., 1987). Differently, in the mR1^Δ/Δ^/R2^Δ/Δ^ mice, albeit the number of myogenin^+^ labeled cells appeared slightly reduced in FGF2-treated vs. untreated myofibers, there was no statistical difference between the two groups.

### FGFR4 Does Not Appear to Substitute for the Mitogenic Effect of FGFR1 on SC Performance in Isolated Myofibers

**FIGURE 8:**
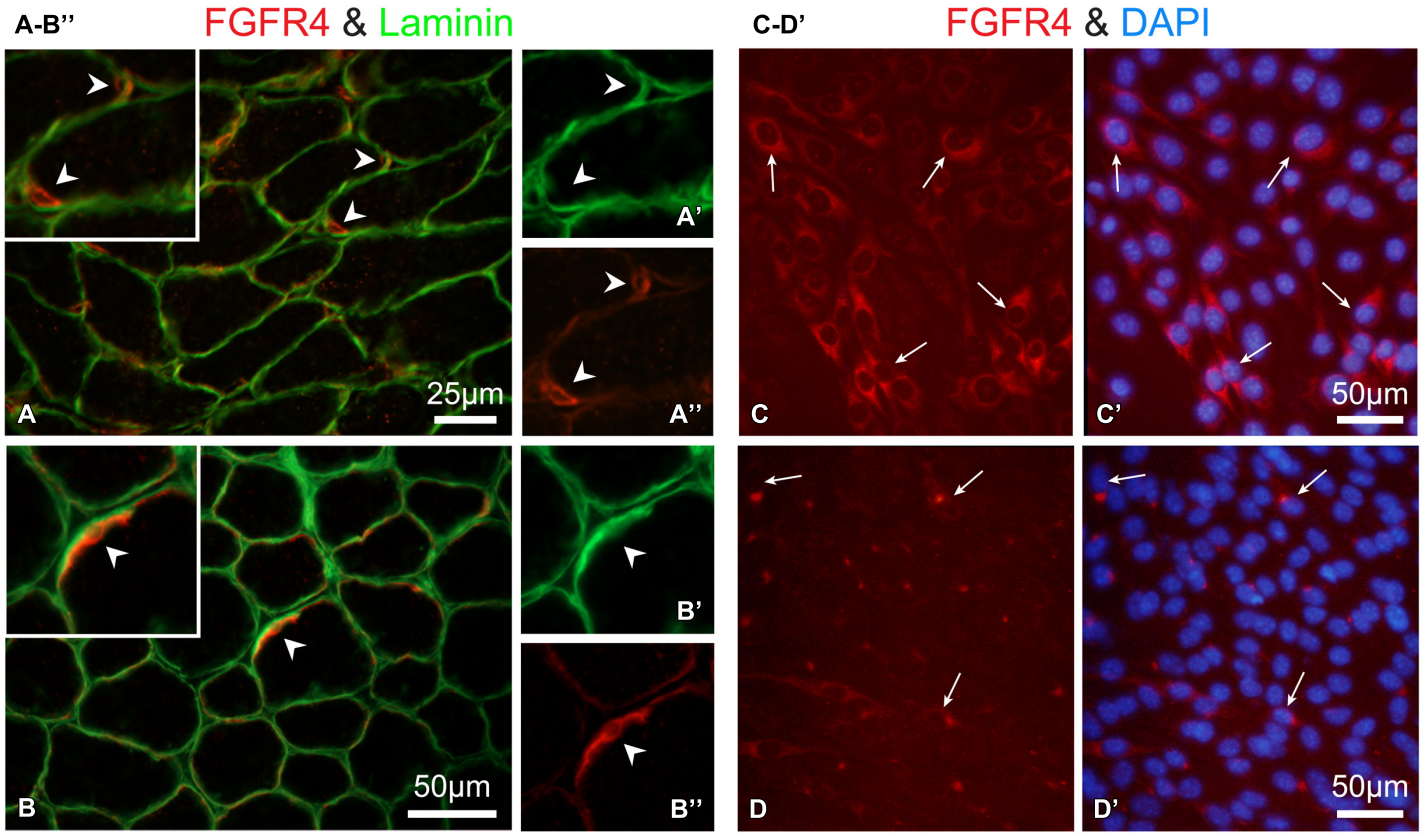
**Immuno-detection of FGFR4 protein in muscle tissue and primary myogenic culture from wildtype mice. (A,B”)** Detection of FGFR4 in hindlimb muscle sections; positive cells are presumptive SCs based on their location underneath the myofiber basal lamina highlighted by laminin immunostaining Notably, SC identification using Pax7 immunostaining is precluded as it would require antigen retrieval step which is not compatible with the conditions used here for FGFR4 detection on unfixed cryosections. As expected, SCs (FGFR4^+^) were more abundant in **(A–A”)** the younger aged mouse (12 days old, gastrocnemius muscle) than in **(B–B”)** the 30-day-old mouse (TA muscle). Corresponding arrowheads denote common locations in the lower and higher magnification images. **(C–D’)** Detection of FGFR4 in primary myogenic cultures from adult mice; the myogenic nature of the cultured cells was verified with double immunostaining for desmin as in (Yablonka-Reuveni et al., 1999a; data not shown). **(C,C’)** FGFR4 protein expression is unique to the myogenic cells while residual non-myogenic cells present in this standard primary culture are negative. **(D,D’)** FGF2 treatment (20 ng/ml in DMEM containing 2% horse serum for 16 hours) of mouse primary myogenic cultures results in the downregulation of FGFR4. Following the overnight treatment with FGF2, FGFR4-immunosignal is restricted to a perinuclear compartment likely reflecting receptor desensitization through its internalization and targeting to endosomes.

Overall, the data in **Figure 6B** demonstrate an impairment of FGF2-mediated proliferative activity of SCs in isolated myofiber cultures from mice lacking functional FGFR1 (and FGFR2). This impairment suggests that other FGFRs that are possibly expressed by SCs cannot substitute for FGFR1 function. As the expression of Fgfr4 transcripts was indeed detected in freshly isolated SCs and their progeny (**Figures 1** and **2**), we set out to determine if FGFR4 protein is expressed by SCs. Previously we and others have shown FGFR4 protein in mouse SC progeny using Western blotting of cultured cells (Kwiatkowski et al., 2008; Cassano et al., 2011). Here, we show immunodetection of FGFR4 in limb muscle cross sections (**Figures 8A–B**”). The observed FGFR4^+^ structures are presumptive SCs based on their location underneath the myofiber basal lamina that is highlighted by laminin immunostaining (**Figures 8A–B**”). We additionally show here the expression of FGFR4 protein in mouse myogenic primary cultures (**Figures 8C,C**’). FGFR4 was down regulated in response to FGF2 supplement, therefore it appears to be functional (**Figures 8D,D**’).

The inability of the endogenously expressed FGFR4 to rescue the proliferative effect of FGF2 in isolated myofibers from mR1^Δ/Δ^/R2^Δ/Δ^ provides further support to our hypothesis that FGFR4 has a different role from that of FGFR1 during adult myogenesis. Indeed, overexpression studies have indicated that different from the other three FGFRs, FGFR4 appears to be a poor inducer of mitogenesis, whereas a clear mitogenic effect was detected when the intracellular domain of overexpressed FGFR4 was replaced with that of FGFR1 (Ornitz et al., 1996; Zhang et al., 2006). The poor mitogenic effect of FGFR4 could be linked to its much reduced tyrosine kinase phosphorylation compared to the other FGFRs (Kwiatkowski et al., 2008). Our FGFR4 overexpression studies [(Kwiatkowski et al., 2008); R Almuly and Z Yablonka-Reuveni, unpublished] have suggested a role for FGFR4 in suppressing FGFR1 tyrosine kinase activity and downstream signaling via FRS2-Erk1/2 axis (Goetz and Mohammadi, 2013), thereby leading cells to withdraw from the cell cycle. Moreover, an earlier FGFR4 overexpression study using L6E9 rat myoblasts demonstrated a weak mitogenic activity for FGFR4 and a role in inhibition of myogenic differentiation (Shaoul et al., 1995). Hence, FGFR4 might provide fine-tuning among proliferation, differentiation and renewal, counteracting the role of FGFR1 in enhancing myoblast proliferation.

## Conclusion

This current study of Fgfr expression profile in freshly isolated SCs and their progeny from adult limb and diaphragm muscles provides new experimental evidence to the commonly held convention that of the four FGFRs, only Fgfr1 and Fgfr4 are of potential relevance to myogenesis. Our earlier work has suggested that these two FGFRs might have different functional roles during adult myogenesis. To begin addressing the possible distinct roles of FGFR1 vs. FGFR4, we employed in the present study a genetic approach relying on the MyoD^Cre^ allele for myogenic-specific ablation of FGFR1 (and FGFR2). Albeit this MyoD^Cre^-driven ablation occurs early during embryogenesis, muscle development does not seem to be overtly impaired in the absence of functional FGFR1 (and FGFR2) based on the intact muscle histology of the adult mR1^Δ/Δ^/R2^Δ/Δ^ mice. Furthermore, cardiotoxin-injured muscle of these mR1^Δ/Δ^/R2^Δ/Δ^ mice showed effective regeneration. However, the SC mitogenic response to FGF2 was drastically repressed in isolated myofiber cultures prepared from the myogenic-specific Fgfr1/Fgfr2-ablated mice. Collectively, our study indicates that FGFR1 is important for FGF2-mediated proliferation of SCs, while the role of the expressed FGFR4 has yet to be resolved. To further address the role of FGFR1 and FGFR4, we are developing genetic models for myogenic-specific ablation of these receptors in growing and aging mice.

## Conflict of Interest Statement

The authors declare that the research was conducted in the absence of any commercial or financial relationships that could be construed as a potential conflict of interest.
